# “How are we going to be able to pull that off?”: staff perspectives on the early implementation of mobile medication units in New York State

**DOI:** 10.1186/s13722-026-00694-y

**Published:** 2026-06-24

**Authors:** Megan Miller, Minna Song, Alexandra Bessler, Kristianny Ruelas-Vargas, David Frank, Samantha J. Harris, Jason B. Gibbons, Ashly E. Jordan, Noa Krawczyk, Brendan Saloner

**Affiliations:** 1 Center for Opioid Epidemiology and Policy, Department of Population Health, NYU Grossman School of Medicine, 180 Madison Avenue, New York, NY 10016 USA; 2 Department of Behavioral and Social Sciences, Brown University School of Public Health, Providence, RI USA; 3 Department of Health Policy and Management, Johns Hopkins University Bloomberg School of Public Health, Baltimore, MD USA; 4 Department of Social and Behavioral Sciences, School of Global Public Health, New York University, New York, NY USA; 5 Program on Regulation, Therapeutics & Law, Division of Pharmacoepidemiology & Pharmacoeconomics, Brigham and Women’s Hospital, Harvard Medical School, Boston, MA USA; 6 New York State Office of Addiction Services and Supports, New York, NY USA; 7 Department of Health Services, Policy and Practice, Brown University School of Public Health, Providence, RI USA

**Keywords:** Methadone, Opioid use disorder, Mobile medication units, Medications for opioid use disorder, Opioid treatment programs, Low threshold treatment, Mobile methadone

## Abstract

**Background:**

Methadone is the gold standard treatment for opioid use disorder (OUD). In the U.S., methadone is usually only available through licensed opioid treatment programs (OTPs), but a 2021 federal rule provided an opportunity for OTPs to provide methadone on mobile medication units (MMUs). MMUs operate under the license of an OTP and are subject to complex regulatory requirements. New York State provided grant funding to support OTPs to adopt MMUs, aligned with the broader goal to improve methadone access statewide. This study explored barriers and facilitators to MMU implementation across New York State from the perspectives of treatment staff and administrators.

**Methods:**

We conducted semi-structured interviews between June 2024 and June 2025 with 16 staff from four OTPs that adopted MMUs and one residential treatment program served by an MMU. Interviews were audio-recorded, transcribed, and analyzed using a hybrid deductive-inductive thematic analysis approach to identify implementation barriers and facilitators.

**Results:**

Staff described a variety of potential models for using MMUs to expand access. In New York City, MMUs were used to serve a residential substance use program. In upstate NY, MMUs were deployed to reduce travel distance in counties with few OTP options. Key facilitators of MMU implementation included leadership persistence in the face of community pushback, creativity and workarounds in the face of logistical hurdles, and support from the state agency. Key barriers included community resistance to MMUs, unclear or inconsistent guidance from the Drug Enforcement Administration, and a variety of operational challenges, such as vehicle maintenance and workforce shortages. Staff generally were positive about the opportunity to use MMUs to address access challenges.

**Conclusions:**

MMUs provide a novel approach to expand methadone access, particularly to populations not currently served by brick-and-mortar OTPs. Early implementers can provide important lessons about how to manage start-up challenges, which can guide later adopters.

**Supplementary Information:**

The online version contains supplementary material available at 10.1186/s13722-026-00694-y.

## Background

The opioid overdose crisis claimed over 80,000 lives in the U.S. and over 4000 lives in New York State in 2024 [1, 2]. Ensuring equitable and sustainable access to methadone, a gold standard treatment for opioid use disorder (OUD), is imperative to addressing the overdose crisis [3, 4]. Methadone has been shown to cut the risk of overdose in half [5] and reduce frequency of illicit opioid use [6], among other health benefits [3, 7, 8]. 

Methadone for OUD is highly regulated in the U.S. and can only be delivered in specialized licensed opioid treatment programs (OTPs), currently available in just 20% of U.S. counties [9]. Travel distance can be a substantial barrier, especially because most patients travel to methadone on a daily basis [10, 11]. In New York State, despite a relatively large number of OTPs, access remains unevenly distributed, with over 50% of counties in Upstate New York lacking an OTP [12]. As a result, patients in these areas may face substantial travel burdens to access methadone treatment, underscoring the need for alternative service delivery models.

One potential way to expand methadone is via mobile medication units (MMUs). MMUs are motor vehicles that have similar rules to a brick-and-mortar OTP but can travel to a variety of locations to dispense methadone and buprenorphine and provide counseling, physical exams, and other health services. In July 2021, the Drug Enforcement Administration (DEA) finalized a rule that allowed OTPs to establish and operate MMUs under their existing licenses [13], lifting a moratorium on MMUs that had been in place since 2007. New York State represents one of the first large-scale statewide efforts to support MMU implementation through targeted funding and policy support.

Research on MMUs conducted before the 2007 moratorium suggests that they can reduce travel burdens, expand methadone access, and reduce overall barriers to OUD treatment [14]. A 2025 report on MMU implementation, which included 8 MMU programs across multiple states in the U.S., found that financing and environmental barriers (e.g., zoning regulations) were major barriers to MMU implementation [15]. Still, MMU implementation remains relatively limited nationwide, with MMUs located in just 17 states. More in-depth research about the real-world challenges and facilitators of MMU implementation, particularly in special populations such as those in residential treatment programs, is needed. To address this gap, we interviewed staff operating and engaging with four MMUs in New York State about facilitators and barriers to successful implementation.

## Methods

1. Study setting.

Compared to other Northeast states, New York State has a large and geographically diverse OTP system, with substantial rural-urban variation in methadone access. These disparities are particularly pronounced in Upstate New York, where patients may face long travel distances to treatment. As a way to increase methadone access, the New York State Office of Addiction Services and Supports (OASAS) – the agency overseeing substance use treatment in New York State—issued 10 competitive grants in 2022 to OTPs to support the adoption of MMUs [16]. This initiative represents one of the earliest coordinated statewide investments in MMU implementation in the U.S. Selected OTPs could choose to expand their reach by offering MMU services to underserved geographic areas with unmet need, including rural regions, or serve populations with limited methadone access, such as patients in residential treatment programs. Our study focuses on the implementation of four MMUs that received OASAS funding.

2. Data Collection.

We conducted semi-structured interviews with staff involved in New York State MMU implementation and the early months of operation. Verbal consent was obtained by the study team before each interview. The study was approved by the Johns Hopkins University Institutional Review Board.

Using a purposive sampling approach, staff from four MMU-implementing OTPs and one residential treatment program partnering with MMU services in New York State were recruited in person during site visits and via email to participate in qualitative interviews. The four MMU sites were selected based on their stage of implementation (i.e., MMUs preparing to launch or in the early stages of MMU operation during the study period). Participating sites reflected variation in geographic location (New York City and Upstate New York), allowing us to capture early implementation experiences across diverse settings.

The study team recruited 13 staff from the OTPs and three staff from the residential treatment program between June 2024 and June 2025. The extended data collection period reflects the staggered rollout and varying implementation timelines across sites, as we conducted interviews when sites were approaching launch or in the early stages of MMU operations. Three of the OTPs with MMUs were in Upstate New York with rural MMU sites, and one OTP and the residential treatment program were in New York City. Each 45–60-minute semi-structured interview was conducted in person or via Zoom by a trained study team member who was not involved in the oversight or funding of participating MMU sites. To support candid responses, interviews were conducted in private settings, participation was voluntary, and participants were assured that responses would be de-identified. Participants received $60 gift cards. The interviews were audio-recorded, transcribed, and de-identified.

The semi-structured interview guide for OTP staff asked about the experience of procuring, financing, launching, and operating MMUs in the early months of the program, while residential treatment program staff were asked about the decision to partner with the MMU, logistics, and impacts on residential program staff and patients. Questions were guided by the Consolidated Framework for Implementation Research (CFIR) [17]. The interview guides included prompts about implementation experiences, including perceived challenges and supports (see Additional Files 1 & 2).

3. Data analysis.

The coding of interviews was conducted by three individuals trained in qualitative coding (MM, AB, MS). An initial codebook was developed based on the interview guide, both of which were informed by CFIR domains. While CFIR informed codebook development, this study was not designed to apply CFIR as a strict analytic framework. Instead, we conducted a thematic analysis to identify barriers and facilitators to MMU implementation, allowing themes to emerge across and beyond CFIR constructs. We used a hybrid deductive-inductive approach, in which some codes were informed by CFIR constructs while additional codes were developed inductively from the data. Four interviews were cross-coded using Dedoose qualitative software to further refine and expand the codebook and the coding team met bi-weekly to discuss and resolve coding discrepancies [18]. Coder agreement was determined by the consensus among the coding team of coding decisions following discussion rather than a formal reliability statistic [19]. After the codebook was finalized, the remaining interviews (*n* = 12) were coded independently by members of the coding team. We then conducted a thematic analysis of coded excerpts to identify key barriers and facilitators to the MMU implementation process in New York State, without restricting themes to predefined CFIR domains. Given that interviews were conducted across 12 months, we considered whether findings varied by timing of data collection. Because participating sites were interviewed at similar stages of MMU implementation and no major policy or operational changes occurred during the study period, we did not observe meaningful differences in themes attributable to interview timing. Variations in responses were therefore more reflective of site-level context than temporal or policy changes during the study period.

## Results

Our sample included 13 women and 3 men (self-reported gender), with most participants (63%) in supervisory roles. While we did not conduct formal comparisons by staff role, administrative participants tended to emphasize system-level issues such as regulatory navigation and funding, whereas service providers more often highlighted operational and patient-facing workflows. Participant representation varied by site, with most sites contributing 3–5 participants each, while one site had only a single participant. Key MMU implementation barriers and facilitators identified by participants are described below.

### Barriers

Participants reported a range of barriers that slowed MMU implementation, including regulatory, operational, community-level, and patient-level challenges.

### Regulatory uncertainty and inconsistent guidancer

Regulatory uncertainty and inconsistent guidance from federal and state agencies were a key barrier to MMU implementation. During the MMU approval process, participants felt there was a lack of clarity and consistency, especially from the DEA, in the regulatory requirements for MMUs:

I don’t believe we’ve gotten a lot of guidance from [the DEA], but they have a lot of requirements. And a lot of requirements are like, ‘You can submit for an exception here. You can submit for an exception here.’ The DEA will define all of these things, but then it’s up to your local DEA office what they will actually be looking for. So, a little more consistency and communication there, I believe, would’ve been better. – OTP Administrator

Additionally, some participants noted a lack of ongoing support from the State agency overseeing the program following initial MMU implementation:

Historically, working with OASAS, what I find the hardest is that ongoing support after you get going. It’s all hands on deck, and then you’re up and going, and it’s like, ‘Ah, onto the next.’ I think sometimes that gets lost with the actual follow-up unless there’s a problem. And then if there’s a problem, knock, knock, knock. They’re there to be able to let you know that there’s a problem. – OTP Administrator

Regulatory challenges were compounded by financial challenges, as several participants highlighted that existing Medicaid reimbursement structures did not account for the unique costs of mobile service delivery. One OTP leader explained:

We need policies around reimbursement. You can’t reimburse mobile health the same way you reimburse brick-and-mortar. So that has to be taken into consideration when you build the rates, the reimbursement rates for these services. – OTP Administrator

### Operational, infrastructure, and workforce constraints

Operational and workforce-related constraints were among the most pervasive barriers, affecting both MMU implementation and sustained service delivery. Participants explained that there were many unique operational challenges with maintaining an MMU vehicle compared to brick-and-mortar operations. In addition to providing their usual services, OTPs must maintain motors, generators, heaters, and other physical components of the vehicle, which adds additional logistical and operational burdens for staff and can occasionally interrupt service delivery. One MMU staff member described an example of maintenance issues they faced:

There has been more maintenance issues than anticipated for only operating a couple of times. To really provide the services we offer on it, we have to slide out the sides [of the MMU] to use the exam room and stuff we have on there. And so the motor has already gone on that twice. – OTP Administrator

Other participants described challenges with finding a place to park their MMU vehicle overnight. DEA regulations required that the MMU be stored in a secure, fenced-in location, which not all OTP programs had access to. One OTP staff member described the process of adding a gate to their parking lot to meet DEA requirements:

[The] DEA just said it needed to be parked in a fenced-in location…if [the MMU] was being placed in our parking lot, we have staff who park there. So I was also thinking of automation and how to make sure that staff will still have access to the parking lot…And then there were a whole lot of other things that popped up like the city’s involvement in the city planners and regulations that the city requires with implementing any kind of changes to property in the city…oh, and then the city needed technical drawings or something. There was going to be an extra cost from the gate company that we were going with. – OTP Administrator

While OTP leaders were excited about providing MMU services, staffing concerns arose for many OTPs. One OTP staff member explained their problem with staffing:

We have our MMU… that’s still not up and running because we don’t have staff for it. It’s not because the mobile medication unit is not ready, they have zero staff for it. – MMU Service Provider

Specifically, not having a physician on board served as a barrier to methadone inductions at the residential treatment program, as described by one of the residential treatment program leaders:

Inductions are difficult because we’re still not doing them on the MMU because of personnel resources. We have to send them to the home clinic. We’re working through that now. We’re doing some virtual work. That’s been a struggle. – Residential Treatment Program Administrator

Before implementation, participants disclosed that some staff were hesitant to work on the MMU. Much of this hesitancy stemmed from uncertainty about how the MMU would operate and a fear of safety related to transporting and storing methadone, a controlled substance, in a vehicle that felt less secure than a brick-and-mortar OTP:

I know there was some concern for having a large amount of methadone on the bus. Some people were concerned about, ‘Well, what if somebody breaks in or tries to steal the methadone? – MMU Service Provider

In the absence of established workflows, initial safety concerns were compounded by uncertainty about day-to-day MMU operations. This lack of clarity limited comfort and buy-in. As one OTP leader explained:

But other staff were like, ‘Oh, I would never work on that [the MMU],’ or, ‘You’re not going to get me on that,’ right? They have made statements like that really out of comfortability. I’m not quite sure if all staff kind of knew what the workflow would look like or what all of the procedures exactly were. So I’m wondering if that was due to a lack of understanding. – OTP Administrator

### Community and stakeholder resistance

Community opposition toward methadone created barriers to MMU site selection, with some participants reporting difficulty in finding a location to operate the MMU. Some OTP staff spoke with officials from local government units^1^ in multiple counties before identifying one that would allow them to operate the MMU:

[We] then identified areas we wanted to go to and then started working at trying to get face time with their local government units. And it was kind of wild. People were really resistant…[the selected county] was after multiple failed attempts in other counties. – OTP Administrator

The opposition from some local government units during MMU implementation reflected persistent stigma toward methadone and resistance to having treatment services in their communities. Specifically, securing local approval for MMUs required negotiation and advocacy to overcome pushback from local governments. One OTP leader explained:

[The] MMU provides services in places where I had to fight to get them there, where elected officials don’t want people who are using methadone to be in their communities, where they don’t want treatment sites in their communities. And so it’s no different from rolling in in a MMU versus a brick-and-mortar. Some communities just don’t want methadone treatment in their communities. – OTP Administrator

### Limited patient demand and acceptability

Demand for MMU services among existing OTP patients was lower than anticipated. Participants noted that patients had varying reasons for wanting to continue traveling to the brick-and-mortar OTP instead of utilizing the MMU, including not wanting to lose access to important social networks. As one OTP staff member described:

I think it’s a transition for those people who just wanted to come into [the brick-and-mortar OTP] because they got used to seeing all of us here every day. So now it’s a change…They don’t get the interaction with all the people that they know that are here at the clinic anymore—even their drug dealers or their friends that come… They don’t get to see their counselors face-to-face. They have to do telehealth now. That’s all a change for people. – MMU Service Provider

### Transportation and structural access barriers

Similar to brick-and-mortar OTPs, transportation challenges for patients emerged as a significant barrier to MMU implementation, particularly in Upstate New York counties where many patients relied on Medicaid-funded transportation to reach the MMU. Participants reported that scheduled rides for patients were often unreliable, with cabs failing to arrive or canceling at the last minute:

In the initial stages of starting up, cabs were just not coming and then canceling appointments and then canceling rides on our patients. And then we didn’t know about it until it was too late. – MMU Service Provider

## Facilitators

Despite barriers, participants also noted several important factors that facilitated MMU implementation, including financial support from OASAS, innovative program cultures and committed leadership, context-specific implementation strategies, and collaboration between service providers.

### State funding support and financial flexibility

One key facilitator was OASAS’s efforts to expand financial support to MMU-implementing OTPs. Participants noted that the initial $200,000 from OASAS was inadequate to realistically fund an MMU, but OASAS found ways to provide more funding to support the OTPs throughout the implementation process:

We got offered some deficit funding from OASAS. I think that helped put our minds at ease a little bit because it’s a five-year thing. So even if next year or the end of this year, we end up being $250,000 in the hole or something, at least that deficit budget pulls us out of that for the next five years. So at least in sustainability in that way, I think we are feeling a little bit better now. – OTP Administrator

### Innovative program culture and proactive leadership

Many of the participants described having a program culture of innovation and novelty with the services they provide, which influenced them to apply for the MMU grant, even if they thought implementation would be difficult:

I would say, first of all, there’s always trepidation with these things, but [our OTP] is, I think, pretty aggressive in terms of taking on challenges and innovative approaches…We don’t really mind being first. – OTP Administrator

Additionally, participants discussed having people in leadership positions who were passionate about expanding services and willing to take on a challenge:

Well, I think we had one, the right people working at [the OTP], in terms of a group of people who are thirsty for challenge and increasing services. And so having just the right people there at the moment to say like, ‘Let’s take this on.’ was key because a lot of– I’ve talked to some other agencies who did not take on any of these contracts, who were like, ‘We’re just waiting to see how this goes for you guys.’ They didn’t want to take the risk. And so I think we had a lot of people who were willing to take the risk and were excited about the idea and also passionate about the expansion of services. – OTP Administrator

While MMUs faced some community pushback during the implementation process, the persistence and proactive engagement of OTP leadership were explained by participants as key to overcoming resistance. OTP leadership invested time in building relationships with communities and used a “soft rollout” strategy to educate stakeholders and foster trust before officially launching the units. One OTP leader described this deliberate approach:

I sat in multiple meetings to make friends with folks before I rolled out. I did a soft rollout and I would advise this to anyone who’s doing this. I did a soft rollout for about six months before I put the unit out on the streets. And that soft rollout was to educate and to get buy-in because I didn’t want to deal with the pressures on my staff to deal with it…So I was in the community. I spoke to people. I invited people to come and see the unit. We put out a press release that it’s coming. We did all the marketing that we could to get community and stakeholder buy-in before we launched. – OTP Administrator

### Context-specific implementation strategies

A key facilitator to MMU implementation was the OTPs’ ability to identify and respond to local gaps in methadone services. OTPs in both New York City and Upstate New York recognized distinct community needs and tailored their implementation strategies accordingly. Given that almost half of counties do not have an OTP, Upstate New York staff viewed MMUs as a way to increase medication for opioid use disorder (MOUD) access points, as highlighted by an OTP staff member:

Access to care has always been my mindset since the day that I’ve stepped into an OTP. And if we could find better ways to improve engagement and improve MOUD services without necessarily having to establish a whole brand new clinic and all the financial components that come along with it. There’s still some counties that don’t have OTPs and this was an opportunity to kind of see if there was a way of promoting the type of services that we’re providing in our brick and mortar in another county without establishing all the processes that you would have to go through in deciding to build a whole brand new clinic. – OTP Administrator

In contrast, New York City-based OTPs leveraged MMUs to reach underserved populations within an already saturated OTP treatment landscape. Rather than adding geographic access points, leaders envisioned bringing methadone to places such as residential facilities where people face barriers to methadone treatment. One OTP leader explained their thought process:

I don’t think in New York City the issue is access points…So the idea was to sort of partner with agencies…and say, ‘If we came to your facility on a regular basis and provided access to methadone, would that increase your ability to take individuals who are utilizing methadone?’ which would then reduce that barrier. – OTP Administrator

### Cross-sector collaboration and care integration

One OTP explicitly formed a partnership with a local residential treatment program to offer methadone services via the MMU. Staff at the residential treatment program attributed the success of the MMU implementation to fostering a strong relationship between programs:

We created almost one-to-one connections, the program director to the assistant vice president, the MOUD coordinator on our side, to the registered nurse on their (MMU) side, the recovery coach on our side to the recovery coach on their side, to establish ongoing communication that facilitated the health of the relationship. And now…we meet as a group to discuss what’s happening. I feel like that’s one of the big successes. And if I were to replicate this, I would point to the relationships that have really facilitated the success of this endeavor. – Residential Treatment Program Administrator

These relationships supported ongoing day-to-day coordination between MMU and residential treatment program staff. One residential treatment program staff member explained:

[MMU staff] are constantly checking in just to see if anybody wants to go up or down on their dose, just to check in to see how they’ve been doing clinically. So having that relationship with the MOUD worker just so we can both coordinate clinically, and medically, just to keep them updated. “Hey, Susie went up 80 milligrams. How has she been while in treatment? Has she been nodding? She’s been craving more.” So, building that relationship with the MOUD worker and the nurse is very beneficial to me because at least I know the information is being filtered and reported. – Residential Treatment Program Service Provider

## Discussion

Our study examined the experiences of treatment program staff involved in MMU implementation across New York State to understand the MMU implementation process following the 2021 DEA rules. MMUs represent one strategy for expanding access to methadone, particularly for patients facing transportation barriers or other structural challenges. They are a part of a broader continuum of low-threshold interventions, such as mobile buprenorphine treatment, aimed at addressing the overdose crisis [21]. Prior studies of mobile methadone and buprenorphine programs have demonstrated their potential to expand access, while also identifying implementation challenges related to regulation, workforce, and community acceptance [15, 22, 23]. However, less is known about how these factors interact during early implementation, particularly in the context of a large-scale statewide effort such as New York State’s early investment in the rollout of multiple MMUs. While MMUs were widely viewed as a promising strategy to reach underserved populations, their implementation in New York State was shaped by the interaction of regulatory, workforce, community, operational, and financial factors, which collectively influenced feasibility, sustainability, and service delivery. A key strength of this study is the inclusion of perspectives from both administrative and service providers, offering a more comprehensive view of MMU implementation.

Consistent with prior mobile MOUD studies, regulatory ambiguity, particularly inconsistent interpretations from local DEA agents, sometimes led to stricter requirements beyond federal regulations [15, 22]. Operational challenges, including vehicle maintenance and associated costs, further constrained service delivery, particularly in regions with long travel distances [14, 15]. Additional procedural requirements, including zoning approvals and infrastructure modifications such as gate installation, introduced unexpected costs and delays. Together, these findings suggest that MMU implementation is influenced by variability in both regulatory interpretation and infrastructure systems, with successful implementation depending on alignment across policy enforcement and the staffing, maintenance, and operational infrastructure required to support mobile service delivery.

Workforce constraints also emerged as a primary barrier, with several OTPs unable to begin operations or consistently operate due to staff shortages. Workforce shortages have been an issue across behavioral health services in the U.S., likely driven in part by burnout and high turnover rates [24, 25]. Prior research has demonstrated that workforce shortages directly constrain the capacity of OUD treatment systems to respond to the opioid crisis, limiting access to care and the ability to scale evidence-based treatments such as methadone [26]. Additionally, studies of methadone treatment staff have identified high levels of trauma-related stress and burnout, which may further impact workforce stability and retention [27]. Hesitancy to work on the MMU, due to perceived safety risks, uncertainty about workflows, and skepticism about the unit’s value, further hindered operations, although prior reports suggest actual safety incidents are minimal [14]. While limited literature directly examines staff safety concerns in MMU-specific settings, one study of mobile opioid treatment units reported higher concerns about medication security and theft risk for a mobile unit than a brick-and-mortar setting [28]. Addressing the gap between perceived and actual safety risks may help secure staff buy-in. Overall findings suggest that mobile delivery models may amplify existing behavioral health workforce constraints by requiring greater flexibility, cross-site coordination, and adaptation to non-traditional care environments.

Community resistance, rooted in stigma toward methadone, required persistent advocacy to secure approval for MMU operations. Participants described several strategies to mitigate resistance, including leadership-led soft rollout, proactive relationship-building with local governments, and inviting community members to tour or engage with the MMU. Studies on the implementation of mobile methadone or buprenorphine units in other contexts have similarly faced community pushback and emphasized the importance of early community engagement [24–26]. These findings underscore that MMU implementation extends beyond clinical and regulatory requirements and also requires buy-in from local communities.

Demand for MMU services from patients was mixed. Some patients were not interested in switching to the MMU from the brick-and-mortar OTP. This finding highlights that while MMUs may reduce structural barriers, psychosocial and social network factors may also influence treatment engagement. Prior research has shown that for some people on methadone, these social networks provide support, structure, and stability in their daily lives; losing access to them can create chaos, disrupt routines, and undermine engagement [29]. Further research is needed to better understand for whom the MMUs are most beneficial and desirable.

OTPs reported that patients continued to face significant transportation challenges. In practice, unreliable or unavailable transportation options, such as missed or canceled Medicaid-funded rides, limited MMU’s intended benefit of reducing travel burdens. These challenges mirror the same transportation barriers that patients face when accessing brick-and-mortar OTPs [30–32], suggesting that MMUs may shift rather than eliminate transportation barriers, particularly in contexts where underlying transportation systems remain unreliable. MMU implementation may also vary by geography, with rural settings likely facing greater challenges related to travel distance and operational costs than urban and suburban areas, which impacts implementation feasibility. MMU implementation strategies, therefore, need to be tailored to local geographic and infrastructural contexts rather than assuming uniform feasibility across settings.

Participants described substantial upfront capital investments required to launch MMUs, including vehicle purchase and retrofitting, as well as infrastructure for medication storage and security. These start-up costs are distinct from ongoing operational expenses such as staffing, maintenance, fuel, and day-to-day service delivery. This distinction has important implications for implementation, as capital costs may limit initial implementation, while ongoing operational costs influence long-term sustainability. Participants noted that existing Medicaid reimbursement structures do not fully account for the higher costs associated with mobile service delivery, creating a persistent gap between reimbursement levels and actual expenses of operating MMUs. In response, participants described state-level efforts to address these challenges, including supplemental start-up funding and enhanced Medicaid reimbursement rates for mobile services implemented by OASAS [33]. 

Despite the challenges identified, certain organizational and collaborative strategies emerged as key facilitators supporting successful MMU implementation. The role of innovative program cultures and committed champions in successful implementation aligns with previous research, which finds that leaders who champion new models of care are essential for successful adoption of novel service delivery approaches [34, 35]. Additionally, this study highlights how service provider collaboration, particularly between OTPs and residential treatment programs, served as a key facilitator. Establishing structured communication channels, aligning workflows, and developing one-to-one staff relationships across agencies helped build trust, minimize conflict, and support continuity of care for patients engaging with the MMU.

Our findings have several actionable implications. Regulators should prioritize consistent guidance and communication to reduce approval delays. OTPs may need to invest in staff training and engagement strategies to address safety perceptions and support workforce retention, as workforce capacity, burnout, and organizational climate are key determinants of successful implementation [26]. State funders should consider dual funding mechanisms that cover both upfront capital costs and ongoing operational expenses, including transportation and maintenance, while ensuring reimbursement models reflect the unique resources needed for mobile services [15]. Community engagement strategies, including soft rollouts, stakeholder education, and local partnerships, are essential to overcome stigma and increase acceptance of MMUs. Finally, patient-centered implementation strategies should consider both structural and psychosocial factors to optimize acceptability and MMU utilization.

This study has several limitations. First, this study is specific to New York State, with a unique geographical and regulatory context. Findings may not be generalizable due to different geography, policy landscapes, and resources. Additionally, our sample comprises only four OTPs, only one of which operated at a residential treatment program, which may limit the applicability of the findings to broader contexts and settings. These four OTPs were also the first to implement MMUs among the ten funded programs, and thus, the findings may reflect the perspectives of only early adopters. While the study captured perspectives from both administrative staff and service providers, most participants held supervisory or leadership roles. As a result, findings may more strongly reflect system-level considerations rather than the day-to-day operational experiences of MMU staff. Finally, although we included participants from multiple sites, variation in the number of interviews per site may have influenced the balance of perspectives represented across settings.

## Conclusion

Together, these findings suggest that MMUs can expand methadone access, but their effectiveness depends on integrated strategies that address regulatory clarity, workforce capacity, community engagement, patient preferences, and financial sustainability. More work is needed to examine patient outcomes, program scalability, cost-effectiveness, as well as how MMUs can be combined with complementary low-threshold interventions to more comprehensively respond to the ongoing overdose crisis.

## Supplementary Information

Below is the link to the electronic supplementary material.

Supplementary Material 1

Supplementary Material 2

## Data Availability

The datasets generated and analyzed during the current study are not publicly available to protect participants.

## Footnotes

1 Local government units are institutional units whose fiscal, legislative, and executive authority extends over the smallest geographical areas distinguished for administrative and political purposes [20].

## Abbreviations

CFIR: Consolidated Framework for Implementation Research
DEA: Drug Enforcement Administration
MMU: Mobile medication unit
MOUD: Medications for opioid use disorder
OASAS: Office of Addiction Services and Supports
OTP: Opioid treatment program
OUD: Opioid use disorder
