# Supplementary material for: “How are we going to be able to pull that off?”: staff perspectives on the early implementation of mobile medication units in New York State
Source: Addict Sci Clin Pract. 2026 Jun 24;21:51. doi: 10.1186/s13722-026-00694-y (PMC13308191; doi:10.1186/s13722-026-00694-y)
Supplement: Supplementary file 2 — Supplementary Material 2 [file 13722_2026_694_MOESM2_ESM.docx]

**INTERVIEW GUIDE**

**Community Service Providers**

Thank you so much for agreeing to participate in this study about mobile medication units in New York State. We really appreciate your time. My name is [NAME] and I am a [ROLE] at [INSTITUTION].

The goal of these interviews is to gather providers’ perspectives and experiences implementing mobile units. We are also interested to hear providers’ thoughts on how best to serve people with opioid use disorder, including what the role for mobile units may or may not be. Your experience and perspective are really important to us.

I want to confirm that you’re okay with being audio recorded. What you say is confidential, which means that we will not identify you or your organization in what we report from these interviews.

**Introduction Questions**

1. Can you tell me where you work?
2. How long have you been at your current place of work?
3. What is your area of training in?
4. What is your role at your current place of work?
5. What county(ies) or cities does [ORGANIZATION] serve?
6. Prior to the current mobile program, did you have any prior experience providing mobile services for people who use drugs?

**Organization and Community Setting**

1. What services does [ORGANIZATION] offer in general?
   1. What about services like case management, housing assistance, childcare, transportation, job training?
   2. Does your organization offer harm reduction services? If so, which services? If not, do you provide active linkages to harm reduction programs?
2. Are any of these services provided for free or on a sliding scale?
   1. If people cannot afford your services, can you describe what options they have to access similar services?
3. What is the geographic area that your program serves? Do any of your clients come from outside of this area? (why?)
4. I want to ask about the clients you serve. Can you describe the range of substance use disorder needs that you encounter? (Probes: demographics, drug of choice, polysubstance, etc.)
   1. What are some of their common substance use service needs? Do you have enough resources in your community to adequately address these? What are some of the things that you think are working? (Probes: What are some examples of things in your community that help meet your clients’ needs or lower barriers to care? What resources are still needed (if you think the needs are not addressed)?)
5. Stigma and discrimination can affect the care that people with OUD receive, or even whether they get care at all. How do you think stigma impacts services and care for people in your community who use substances and/or who have OUD?
6. What are community perceptions of methadone (how about at your organization? Racial differences in perceptions of methadone?)? What role does stigma play?
7. What about racial discrimination? How do you see that showing up for patients with OUD in your community?

**Mobile Methadone Program**

1. Switching gears, I’d like to talk more specifically about the mobile medication unit program. What populations were the mobile medication unit designed to serve?
   1. How do you think mobile methadone will affect people who don’t access or have trouble accessing a fixed OTP?
2. [Management only] Can you describe the process of decision making that led to being involved in the mobile methadone planning process or being a stop on the van’s route?
3. [Management only] How did officials from the state or local government help you in this process?
4. Which agencies have you interacted with?
5. What technical assistance have they offered you?
6. What services, information, or assistance have you wanted but not been able to get from the government?
7. [Management only] Who is at the table when decisions are being made about the mobile methadone program? To what extent were current or potential patients involved? Which individuals within and outside your organization have been instrumental in pushing the program forward? Which individuals have been opposed to the program? Please give some examples for both. Who was supportive of the program and why?
8. How do OTP staff or community attitudes influence expansion of mobile methadone?
9. Have you had conversations with law enforcement in the areas where the units are being routed?
   1. What has been the reaction of police? Staff? Participants? What was the resolution of the police interaction?
10. To your knowledge, how do DEA policies impact the implementation of mobile methadone?
11. What other local, State, or Federal policies do you believe influence the implementation of mobile methadone?
12. If you could change one thing about the mobile methadone program to improve care for people with OUD in the community, what would it be?
13. What do you see as the main unresolved challenges related to methadone access and retention? (Probes: How will the state know if it has succeeded in its goals? Do you have recommendations for other states looking to implement mobile methadone programs?)

**Future Steps**

1. What additional services or steps are needed to ensure that mobile methadone is effective at meeting the needs of the target population?
2. What data would show that it is effective and worthwhile?

**Wrap-Up**

1. That’s all of my questions. Is there anything we didn’t cover that you’d like to talk about?
